# Supplementary material for: Initiation of lumen formation from junctions via differential actomyosin contractility regulated by dynamic recruitment of Rasip1
Source: Nat Commun. 2024 Nov 9;15:9714. doi: 10.1038/s41467-024-54143-y (PMC11550478; doi:10.1038/s41467-024-54143-y)
Supplement: Supplementary file 1 — Supplementary Information [file 41467_2024_54143_MOESM1_ESM.pdf]

Supplementary information

Initiation of Lumen Formation from Junctions via Differential Actomyosin Contractility Regulated by Dynamic Recruitment of Rasip1

Jianmin Yin, Niels Schellinx, Ludovico Maggi, Kathrin Gundel, Cora Wiesner, Maria Paraskevi Kotini, Minkyoung Lee, Li-Kun Phng, Heinz-Georg Belting and Markus Affolter

Contents:

Figure S1, related to Figure 1
Figure S2, related to Figure 3
Figure S3, related to Figure 4
Figure S4, related to Figure 5
Figure S5, related to Figure 6
Figure S6, related to Figure 6
Figure S7, related to Figure 7
Figure S8, related to Figure 7
Figure S9, related to Figure 8
Figure S10, related to Figure 9
Figure S11, related to Method

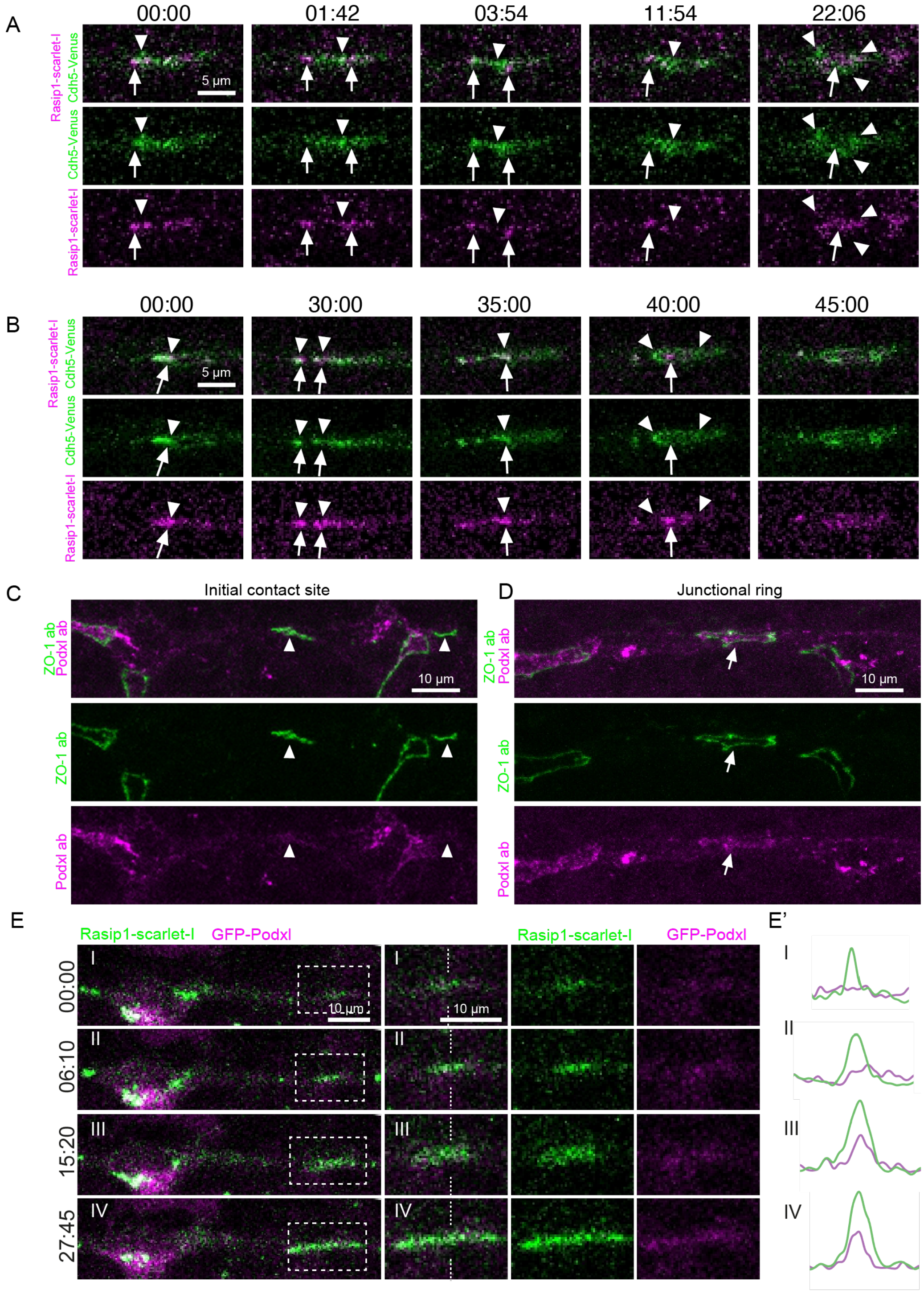

Figure S1. Recruitment of Rasip1 and Podxl1 during the patch-to-ring transition, related to Figure 1

(A and B) Time lapses showing the recruitment of Rasip1 to the junctional patches and nascent junctional rings. Rasip1 translocated to the periphery of the junctional patches. White arrowheads label Cdh5 whilst white arrows label Rasip1.

(C and D) Antibody staining of ZO-1 and Podxl at junctional patches (C) or nascent junctional rings (D). White arrowheads label the junctional patches whilst white arrows label the nascent junctional rings formed by anastomosis. Consistent observations in 12 samples from 3 independent experiments.

(E) Time lapses showing the recruitment of GFP-Podxl to the apical compartments together with Rasip1-scarlet-I. (E') Intensities of Rasip1-Scarlet-I and GFP-Podxl along the dashed lines in (E).

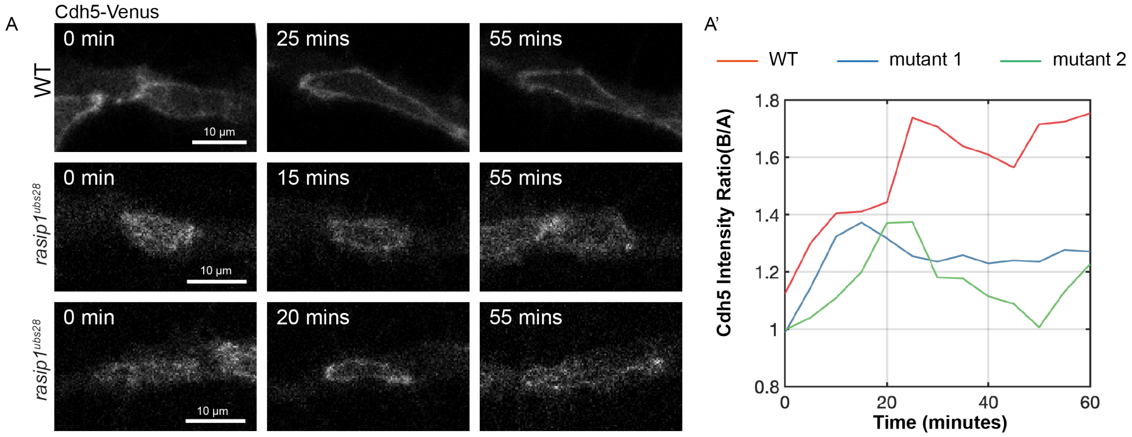

Figure S2. Cdh5 clusters detach from junctions and move towards the apical compartments in *rasip1*^{ubs28} mutants, related to Figure 3

(A) Time lapses of Cdh5-Venus in WT embryos and *rasip1*^{ubs28} mutants. The junctional patches transited into rings transiently but collapsed soon in *rasip1*^{ubs28} mutants. (A') Temporal profiles of the Cdh5 boundary-to-apical ratio in WT embryos and *rasip1*^{ubs28} mutants in (A). Mutant 1: middle panel of (A); Mutant 2: bottom panel of (A). Consistent observations in 35 samples from 5 independent experiments. Source data are provided as a Source Data file.

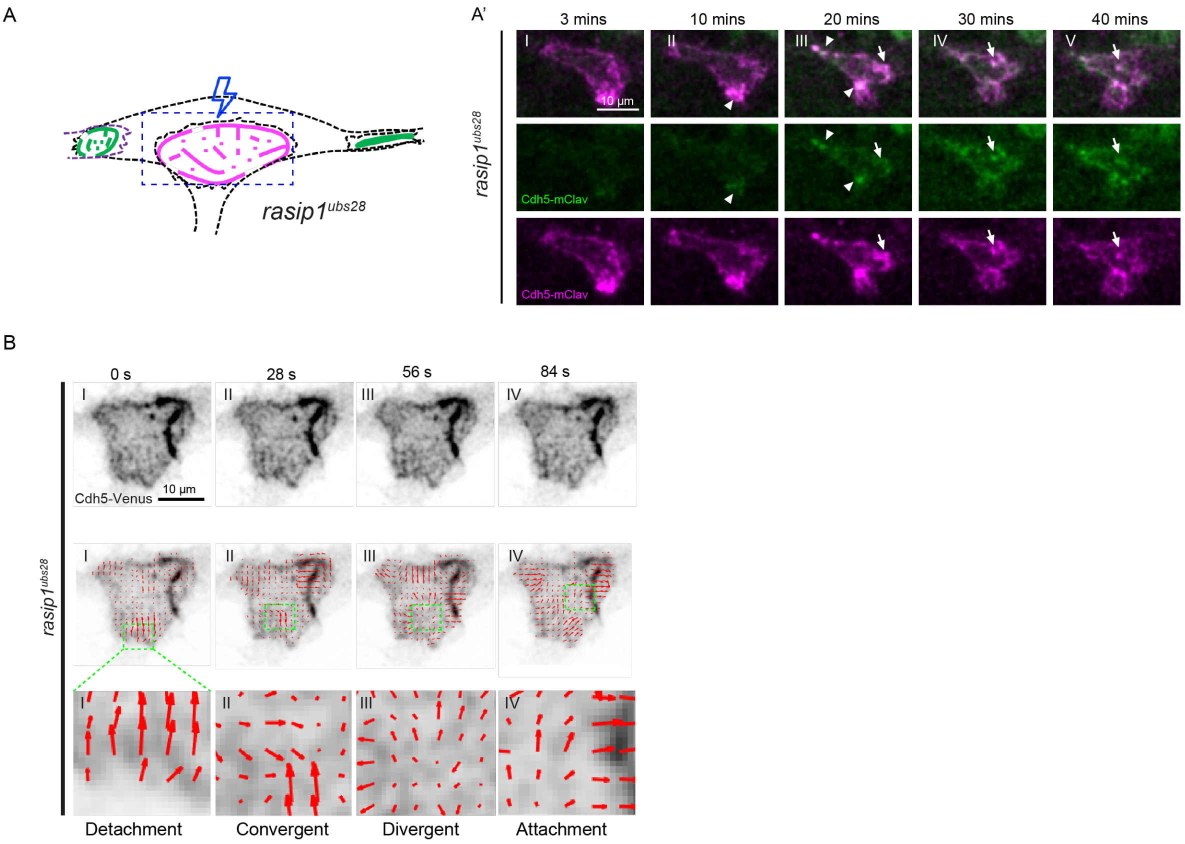

Figure S3. Dynamics of Cdh5 measured by photo-conversion and PIV, related to Figure 4

(A and A') Diagram and time-lapse of photo-conversion on the junctional ring between tip and stalk cells in *rasip1*^{ubs28} mutants. Photo-conversion was performed before the degeneration of the junctional ring into reticulated junctions. White arrowheads label newly incorporated green Cdh5-mClav at the boundaries. White arrows label the Cdh5 clusters moving towards the apical compartment.

(B)Time lapse of Cdh5-Venus in *rasip1*^{ubs28} mutants and corresponding quiver plots generated by PIV showing different patterns of Cdh5 rearrangement at the apical compartments.

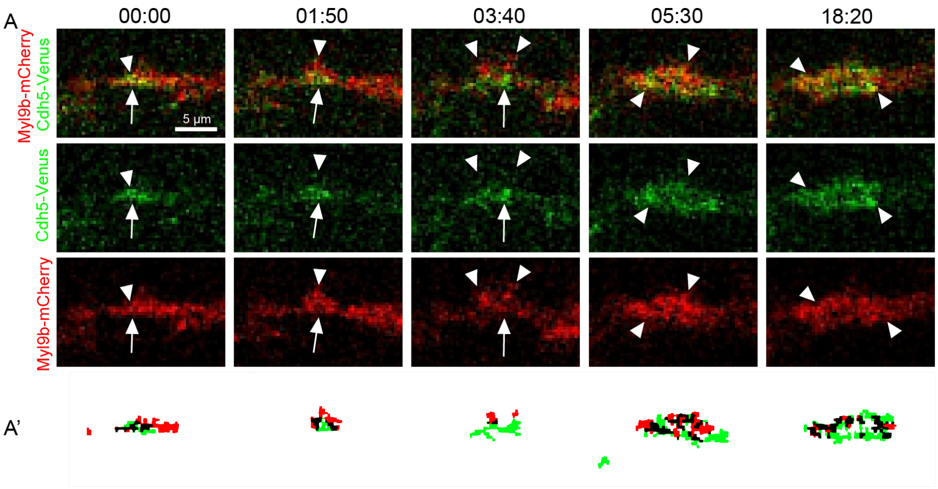

Figure S4. Rearrangement of Myl9 and Cdh5, related to Figure 5

(A)Time lapses of Cdh5-Venus and Myl9b-mCherry showing the rearrangement of Myl9b through the patch-to-ring transition. White arrowheads label Myl9b clusters whilst white arrows label Cdh5 patches. (A') Automatic thresholding of Myl9b-mCherry and Cdh5-Venus in (A). Consistent results were observed in 6 samples.

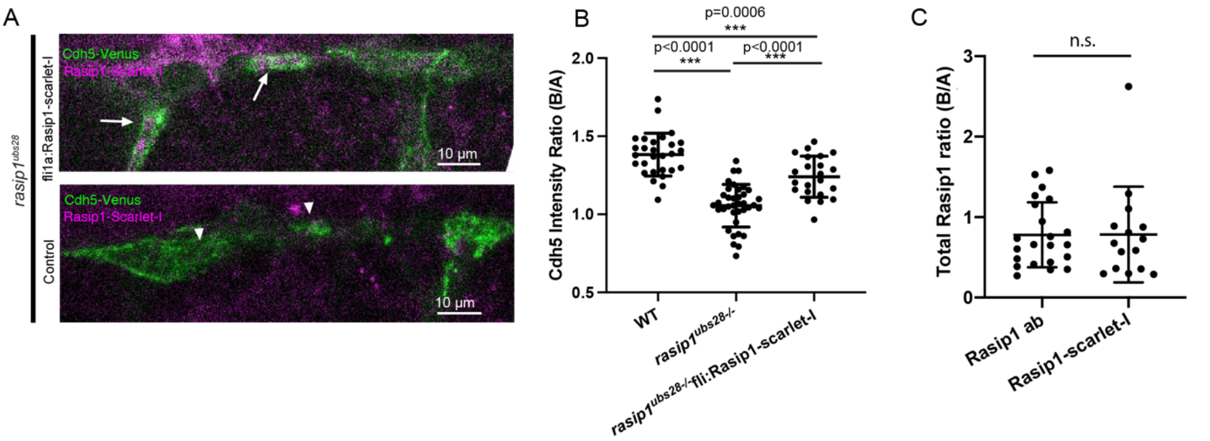

Figure S5. Rasip1 reporters rescued the apical clearance defects and displayed a similar pattern of localization compared to antibody staining, related to Figure 6

(A and B) Expression of Rasip1-scarlet-I under *fli1a* promoter rescued the apical clearance phenotype in *rasip1*^{ubs28} mutants. White arrows label the opened junctional rings with the expression of Rasip1-scarlet-I. White arrowheads label the unopened or reticulated junctions in the absence of Rasip1-scarlet-I in *rasip1*^{ubs28} mutants.

(B) Quantification of the boundary-to-apical ratio of the average Cdh5 intensity (WT: n=28 cells from 8 embryos; *rasip1*^{ubs28}: n=37 cells from 11 embryos; *rasip1*^{ubs28} +Rasip1-scarlet-I: n=22 cells from 6 embryos). Data are presented as mean ± SD. 2-tailed unpaired t-test.

(C) Quantification of the boundary-to-apical ratio of the total amount of Rasip1 in each compartment based on Rasip1 antibody or the Rasip1 live reporter. N.s., not significant, 2-tailed unpaired t-test. Source data are provided as a Source Data file.

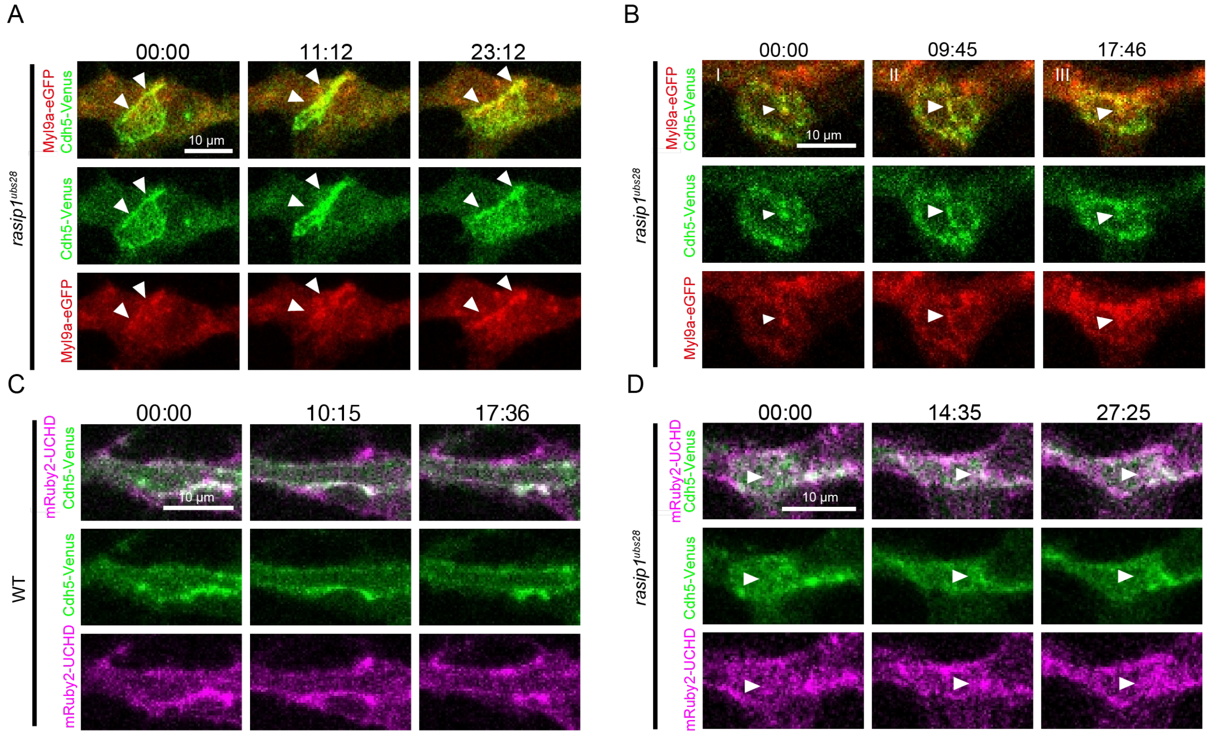

Figure S6. Persisted enrichment of Myl9a and actin in *rasip1* mutants, related to Figure 6

(A and B) Persisted excessive enrichment of Myl9a-GFP at junctions (A) or within the apical compartments (B) in *rasip1*^{ubs28} mutants labelled with white arrowheads. Consistent observations in 14 samples from 5 independent experiments.

(C) UCHD labeled actin primarily localized along junctions in wild-type embryos.

(D) Excessive enrichment of UCHD-labeled actin at the apical compartments of *rasip1*^{ubs28} mutants, indicated by white arrowheads. Consistent observations in 9 samples from 3 independent experiments.

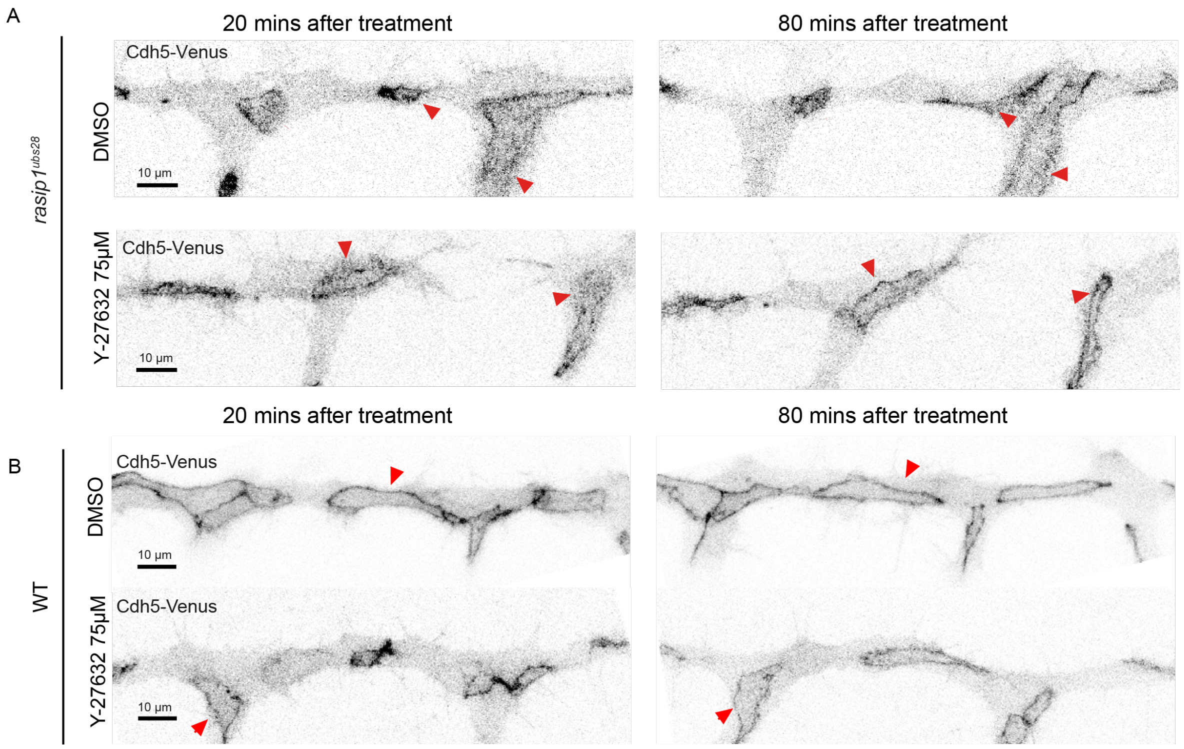

Figure S7. Inhibition of ROCK alleviates the ectopic apical Cdh5 in *rasip1*^{ubs28} mutants, related to Figure 7

(A) Cdh5-Venus at 20 minutes and 80 minutes after 1% DMSO or Y-27632(75 µM) treatment in *rasip1*^{ubs28} mutants. Red arrowheads exhibit the same junctions at 20 minutes and 80 minutes after drug treatment.

(B) Cdh5-Venus at 20 minutes and 80 minutes after 1% DMSO or Y-27632(75 µM) treatment in WT embryos. Red arrowheads exhibit the same junctions at 20 minutes and 80 minutes after drug treatment.

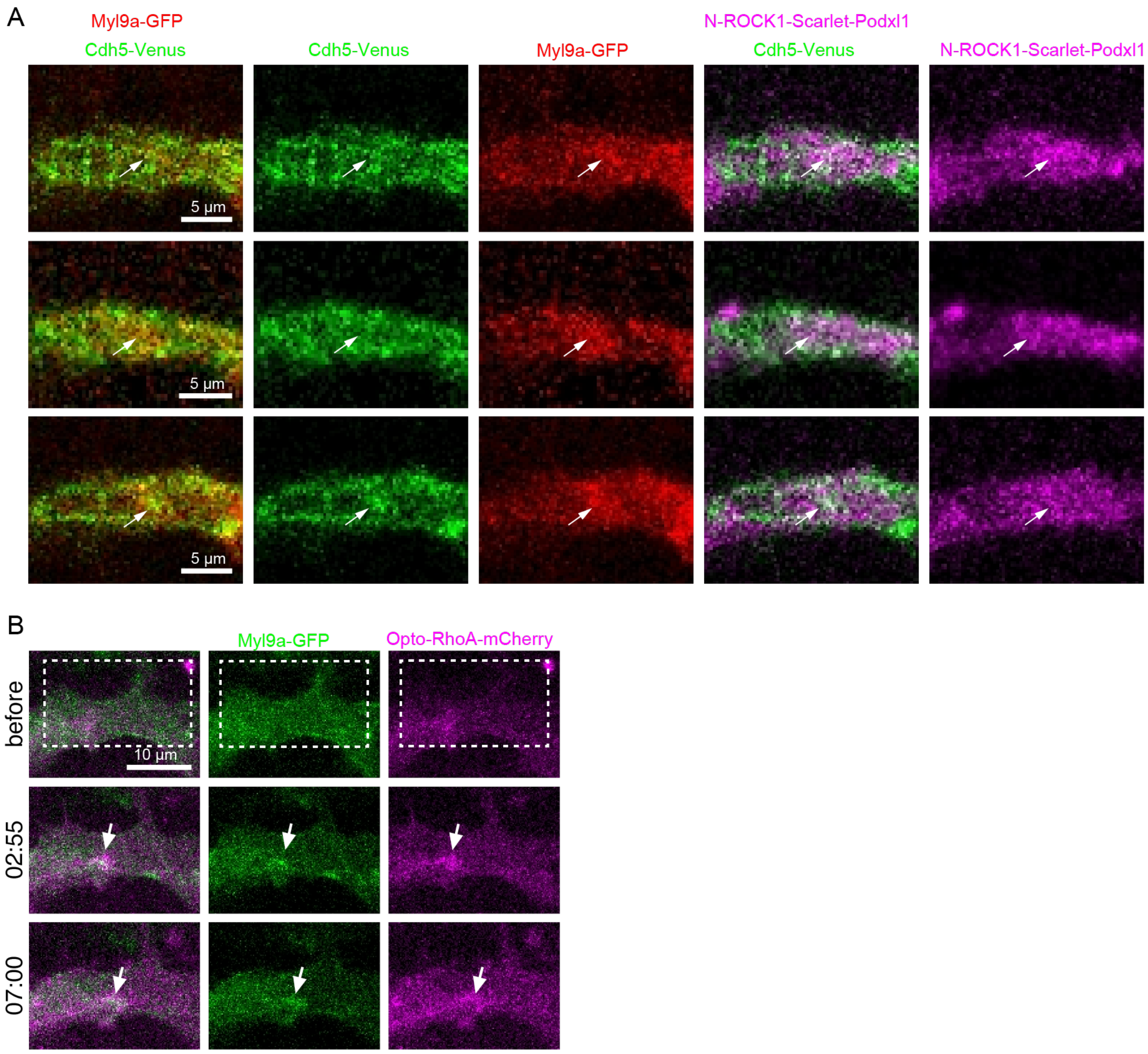

Figure S8. Activation of contractility under Rock1-Scarlet-I-Podxl1 and opto-RhoA-Cherry, related to Figure 7

(A) Wild-type embryos with the expression of Cdh5-Venus, Myl9a-GFP and N-Rock1-Scarlet-I-Podxl1. White arrows label enriched Myl9a-GFP and reticulated junctions at the apical compartments under expression of N-Rock1-Scarlet-I-Podxl1.

(B) Activation of opto-RhoA induced Myl9a-GFP enrichment. White arrows label the colocalized Myl9a and opto-RhoA-Cherry clusters. White dashed lines label the region to be activated by blue laser.

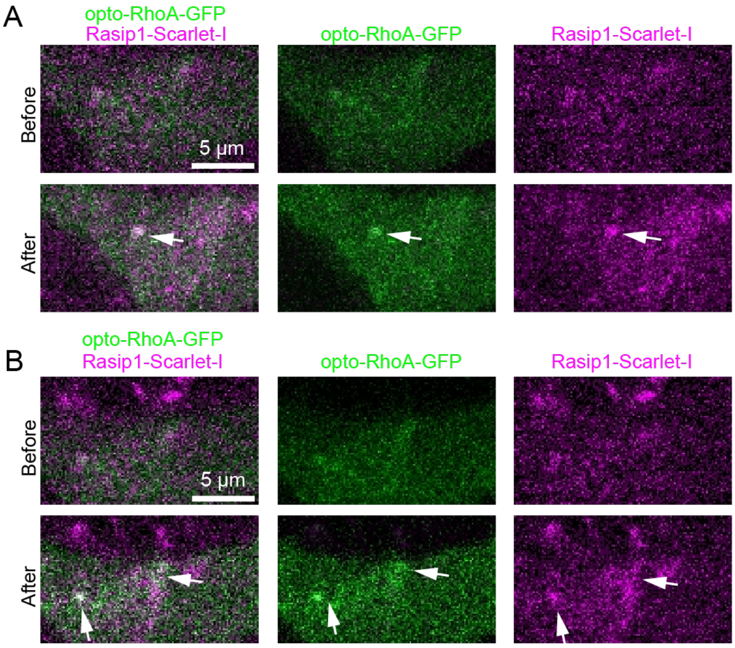

Figure S9. Activation of opto-RhoA-GFP indcues recruitment of Rasip1, related to Figure 8

(A and B) Activation of opto-RhoA induced Rasip1-Scarlet-I enrichment towards the opto-RhoA-GFP clusters. White arrows indicate the colocalized Rasip1-scarlet-I and opto-RhoA-GFP clusters.

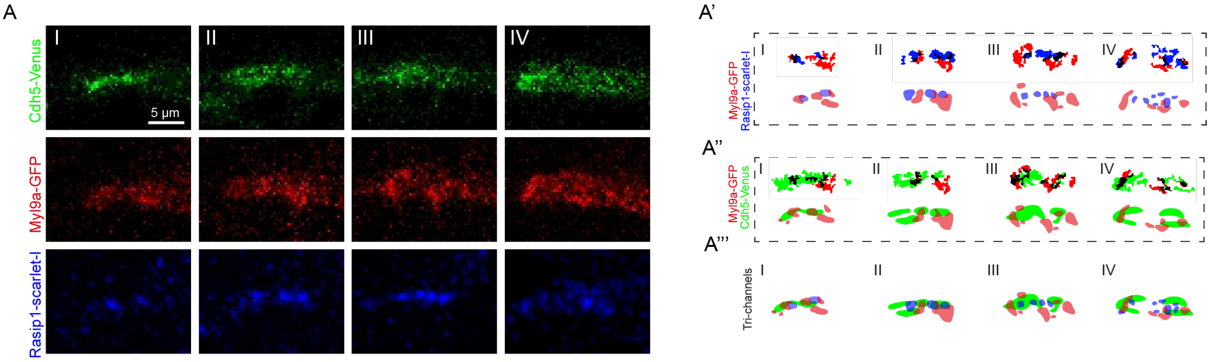

Figure S10. Tri-channels imaging of Rasip1, Myl9a and Cdh5, related to Figure 9

(A) Time lapse of three-color live imaging showing the rearrangement of Cdh5, Myl9a and Rasip1 during *de novo* lumen formation. (A') Automatic thresholding and diagrams showing the localization of Myl9a and Rasip1 clusters. (A'') Automatic thresholding and diagrams showing the localization of Myl9a and Cdh5 clusters. (A''') Graphic diagrams showing the segregation between Cdh5, Myl9a and Rasip1 during *de novo* lumen formation.

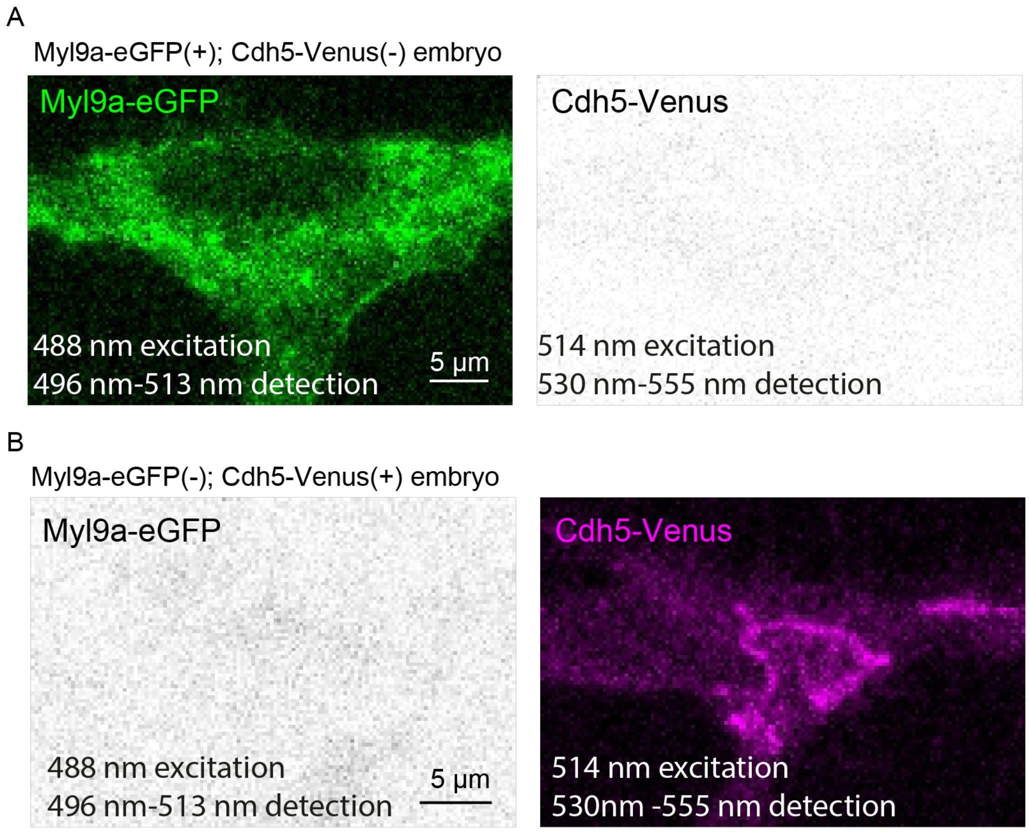

Figure S11. Test of possible crosstalk during live imaging of Myl9a-eGFP and Cdh5-Venus, related to Method

(A) Dual-channel live imaging of embryos expressing only Myl9a-GFP. GFP was excited by 514 nm laser and detected at the range between 496 nm and 513 nm. Venus was excited by 514 nm laser and detected at the range between 530 nm and 555 nm.

(B) Dual-channel live imaging of embryos expressing only Cdh5-Venus.
