# Supplementary material for: Initiation of lumen formation from junctions via differential actomyosin contractility regulated by dynamic recruitment of Rasip1
Source: Nat Commun. 2024 Nov 9;15:9714. doi: 10.1038/s41467-024-54143-y (PMC11550478; doi:10.1038/s41467-024-54143-y)
Supplement: Supplementary file 3 — Description of Additional Supplementary Files [file 41467_2024_54143_MOESM3_ESM.pdf]

Description of Additional Supplementary Files

File Name: Supplementary Movie 1

Description: Anastomosis and *de novo* lumen formation in zebrafish DLAV.

Time-lapse series with expression of Cdh5-Venus (green) and GFP-Podxl1 (magenta) imaged from 30 hpf,, corresponding to Figure 1A.

File Name: Supplementary Movie 2

Description: Dynamics of junctional materials during the patch-to-ring transition.

Time-lapse series with expression of Cdh5-Venus (cyan) and ZO1-Tdtomato (red) imaged from 30 hpf, corresponding to Figure 1B.

File Name: Supplementary Movie 3

Description: Recruitment of Rasip1 to the junctional patches.

Time-lapse series with expression of Cdh5-Venus (green) and Rasip1-scarlet-I (magenta) imaged from 30 hpf, corresponding to Figure 1E.

File Name: Supplementary Movie 4

Description: Degenerations of junctional rings and lumens in *rasip1*^{ubs28} mutants.

Time-lapse series with expression of Cdh5-Venus (green) and GFP-Podxl1 (magenta) imaged from 32 hpf, corresponding to Figure 2I.

File Name: Supplementary Movie 5

Description: Photo-conversions on half junctional rings in WT embryos.

Time-lapse series with unconverted Cdh5-mClav (green) and converted Cdh5-mClav (magenta) imaged from 32 hpf, corresponding to Figure 3E'.

File Name: Supplementary Movie 6

Description: Photo-conversions on half boundary regions of the apical compartments in *rasip1*^{ubs28} mutants.

Time-lapse series with unconverted Cdh5-mClav (green) and converted Cdh5-mClav (magenta) imaged from 32 hpf, corresponding to Figure 3G'.

File Name: Supplementary Movie 7

Description: Cdh5 dynamics in WT embryos.

Time-lapse series with expression of Cdh5-Venus imaged from 32 hpf, corresponding to Figure 4A.

File Name: Supplementary Movie 8

Description: Cdh5 clusters in the apical domains in *rasip1*^{ubs28} mutants.

Time-lapse series with expression of Cdh5-Venus imaged from 32 hpf, corresponding to Figure 4B.

File Name: Supplementary Movie 9

Description: Linear Cdh5 fragments in the apical domains in *rasip1*^{ubs28} mutants.

Time-lapse series with expression of Cdh5-Venus imaged from 32 hpf, corresponding to Figure 4C.

File Name: Supplementary Movie 10

Description: Automatic tracking of Cdh5 clusters in *rasip1*^{ubs28} mutants through PIV.

Time-lapse series with expression of Cdh5-Venus imaged from 32 hpf and corresponding quiver plots, corresponding to Figure S3B.

File Name: Supplementary Movie 11

Description: Dynamics of Myl9a-GFP during the patch-to-ring transition in WT embryos.

Time-lapse series with expression of Cdh5-Venus (green) and Myl9a-GFP (red) imaged from 30 hpf, corresponding to Figure 5D.

File Name: Supplementary Movie 12

Description: Dynamics of Myl9a-GFP during the failed patch-to-ring transition in *rasip1*^{ubs28} mutants.

Time-lapse series with expression of Cdh5-Venus (green) and Myl9a-GFP (red) imaged from 30 hpf, corresponding to Figure 5E.

File Name: Supplementary Movie 13

Description: Dynamics of Myl9a-GFP in the apical compartment and junctions of WT embryos.

Time-lapse series with expression of Cdh5-Venus (green) and Myl9a-GFP (red) imaged from 32 hpf, corresponding to Figure 5I.

File Name: Supplementary Movie 14

Description: Dynamics of Myl9a-GFP in the apical compartment and junctions of *rasip1*^{ubs28} mutants.

Time-lapse series with expression of Cdh5-Venus (green) and Myl9a-GFP (red) imaged from 32 hpf, corresponding to Figure 5J.

File Name: Supplementary Movie 15

Description: Enrichment of Rasip1 at the constricting junctions and apical domains.

Time-lapse series with expression of Cdh5-Venus (green) and Rasip1-scarlet-I (magenta) imaged from 32 hpf, corresponding to Figure 6D and 6E.

File Name: Supplementary Movie 16

Description: Relocation of Rasip1 from the apical domains to the boundary (junctions).

Time-lapse series with expression of GFP-Rasip1 (magenta) and UCHD-mRuby (green) imaged from 32 hpf, corresponding to Figure 6F.

File Name: Supplementary Movie 17

Description: Relocation of Rasip1 from boundary to the apical domain.

Time-lapse series with expression of GFP-Rasip1 (magenta) and UCHD-mRuby (green) imaged from 32 hpf, corresponding to Figure 6G.

File Name: Supplementary Movie 18

Description: Rasip1 clusters colocalized with Myl9a clusters along junctions.
Time-lapse series with expression of Myl9a-GFP (green) and Rasip1-scarlet-I (magenta) imaged from 32 hpf, corresponding to Figure 6I.

File Name: Supplementary Movie 19

Description: Rasip1 clusters colocalized with Myl9a clusters within the apical compartments.
Time-lapse series with expression of Myl9a-GFP (green) and Rasip1-scarlet-I (magenta) imaged from 32 hpf, corresponding to Figure 6J.

File Name: Supplementary Movie 20

Description: Activation of opto-RhoA selectively at the apical compartment induced junction detachment in WT embryos.
Time-lapse series with expression of Cdh5-Venus (green) and RhoA-BcLOV4-mCherry (magenta) imaged from 32 hpf. Blue labels the ROI of activation, corresponding to Figure 7H.

File Name: Supplementary Movie 21

Description: Rasip1-Scarlet-I was poorly enriched along junctions in *krit1* mutants.
Time-lapse series with expression of Cdh5-Venus (green) and Rasip1-scarlet-I (magenta) imaged from 32 hpf, corresponding to Figure 8E.
