# Supplementary material for: Initiation of lumen formation from junctions via differential actomyosin contractility regulated by dynamic recruitment of Rasip1
Source: Nat Commun. 2024 Nov 9;15:9714. doi: 10.1038/s41467-024-54143-y (PMC11550478; doi:10.1038/s41467-024-54143-y)
Supplement: Supplementary file 25 — Reporting Summary [file 41467_2024_54143_MOESM25_ESM.pdf]

nature portfolio

Corresponding author(s): Jianmin YIN

Last updated by author(s): Oct 23, 2024

# Reporting Summary

Nature Portfolio wishes to improve the reproducibility of the work that we publish. This form provides structure for consistency and transparency in reporting. For further information on Nature Portfolio policies, see our Editorial Policies and the Editorial Policy Checklist.

## Statistics

For all statistical analyses, confirm that the following items are present in the figure legend, table legend, main text, or Methods section.

| n/a | Confirmed | |
|---|---|---|
| ☐ | ☒ | The exact sample size ($n$) for each experimental group/condition, given as a discrete number and unit of measurement |
| ☐ | ☒ | A statement on whether measurements were taken from distinct samples or whether the same sample was measured repeatedly |
| ☐ | ☒ | The statistical test(s) used AND whether they are one- or two-sided<br>*Only common tests should be described solely by name; describe more complex techniques in the Methods section.* |
| ☒ | ☐ | A description of all covariates tested |
| ☐ | ☒ | A description of any assumptions or corrections, such as tests of normality and adjustment for multiple comparisons |
| ☐ | ☒ | A full description of the statistical parameters including central tendency (e.g. means) or other basic estimates (e.g. regression coefficient) AND variation (e.g. standard deviation) or associated estimates of uncertainty (e.g. confidence intervals) |
| ☐ | ☒ | For null hypothesis testing, the test statistic (e.g. $F$, $t$, $r$) with confidence intervals, effect sizes, degrees of freedom and $P$ value noted<br>*Give P values as exact values whenever suitable.* |
| ☒ | ☐ | For Bayesian analysis, information on the choice of priors and Markov chain Monte Carlo settings |
| ☒ | ☐ | For hierarchical and complex designs, identification of the appropriate level for tests and full reporting of outcomes |
| ☒ | ☐ | Estimates of effect sizes (e.g. Cohen's $d$, Pearson's $r$), indicating how they were calculated |

*Our web collection on statistics for biologists contains articles on many of the points above.*

## Software and code

Policy information about availability of computer code

| | |
|---|---|
| Data collection | Data were collected through live imaging of zebrafish embryos using confocal microscopy Zeiss SP5 with 40x water immersion objective, focusing on the dynamic recruitment of Rasip1 and organizations of actomyosin and junctions during de novo lumen formation. Immunostaining was performed to label key junctional and apical proteins including Cdh5, ZO-1 and Rasip1. Data were analyzed using imageJ 2.9.0. and MATLAB 2023b with custom scripts, and all experiments were conducted with at least three biological replicates for each condition. The code utilized for data analysis in this study is available on Code Ocean : https://doi.org/10.24433/CO.0511631.v1. |
| Data analysis | GraphPad Prism 9 and MATLAB 2023b were used to perform statistical analysis. Statistical significance was assessed with two-tailed Student's t-tests. Fluorescence intensities from live imaging were quantified using imageJ 2.9.0. or MATLAB 2023b , and results were normalized to control conditions. Error bars represent the Standard deviation, and p-values < 0.05 were considered statistically significant. Experiments were performed with at least three biological replicates. |

For manuscripts utilizing custom algorithms or software that are central to the research but not yet described in published literature, software must be made available to editors and reviewers. We strongly encourage code deposition in a community repository (e.g. GitHub). See the Nature Portfolio guidelines for submitting code & software for further information.

## Data

Policy information about availability of data

All manuscripts must include a data availability statement. This statement should provide the following information, where applicable:

- Accession codes, unique identifiers, or web links for publicly available datasets
- A description of any restrictions on data availability
- For clinical datasets or third party data, please ensure that the statement adheres to our policy

All data supporting the findings of this study are available on Switchdrive: https://drive.switch.ch/index.php/s/UpAJZDe0zVwt8oF. The datasets include Z-projected images, processed data, and analysis scripts. Raw imaging files can be obtained from the corresponding author upon reasonable request. Source data are provided within this paper.

## Research involving human participants, their data, or biological material

Policy information about studies with human participants or human data. See also policy information about sex, gender (identity/presentation), and sexual orientation and race, ethnicity and racism.

| | |
|---|---|
| Reporting on sex and gender | *Use the terms sex (biological attribute) and gender (shaped by social and cultural circumstances) carefully in order to avoid confusing both terms. Indicate if findings apply to only one sex or gender; describe whether sex and gender were considered in study design; whether sex and/or gender was determined based on self-reporting or assigned and methods used. Provide in the source data disaggregated sex and gender data, where this information has been collected, and if consent has been obtained for sharing of individual-level data; provide overall numbers in this Reporting Summary. Please state if this information has not been collected. Report sex- and gender-based analyses where performed, justify reasons for lack of sex- and gender-based analysis.* |
| Reporting on race, ethnicity, or other socially relevant groupings | *Please specify the socially constructed or socially relevant categorization variable(s) used in your manuscript and explain why they were used. Please note that such variables should not be used as proxies for other socially constructed/relevant variables (for example, race or ethnicity should not be used as a proxy for socioeconomic status). Provide clear definitions of the relevant terms used, how they were provided (by the participants/respondents, the researchers, or third parties), and the method(s) used to classify people into the different categories (e.g. self-report, census or administrative data, social media data, etc.) Please provide details about how you controlled for confounding variables in your analyses.* |
| Population characteristics | *Describe the covariate-relevant population characteristics of the human research participants (e.g. age, genotypic information, past and current diagnosis and treatment categories). If you filled out the behavioural & social sciences study design questions and have nothing to add here, write "See above."* |
| Recruitment | *Describe how participants were recruited. Outline any potential self-selection bias or other biases that may be present and how these are likely to impact results.* |
| Ethics oversight | *Identify the organization(s) that approved the study protocol.* |

Note that full information on the approval of the study protocol must also be provided in the manuscript.

# Field-specific reporting

Please select the one below that is the best fit for your research. If you are not sure, read the appropriate sections before making your selection.

☒ Life sciences ☐ Behavioural & social sciences ☐ Ecological, evolutionary & environmental sciences

For a reference copy of the document with all sections, see nature.com/documents/nr-reporting-summary-flat.pdf

# Life sciences study design

All studies must disclose on these points even when the disclosure is negative.

| | |
|---|---|
| Sample size | Sample size was not pre-determined using statistical methods. Later for quantification, minimal sample sizes were calculated based on a power analysis, ensuring that the study had sufficient power (80%) to detect statistically significant differences in apical compartments and cell-cell junction phenotypes between rasip1, heg1, krit1, and cdh5 mutants and controls with a significance level of 0.05. |
| Data exclusions | All data meeting the quality control criteria were included in the analyses. |
| Replication | Immunostaining for Rasip1 and Cdh5, as well as Rasip1 and Myl9a-GFP were successfully replicated at least 4 times in independent experimental setups. Live imaging experiments, involving Rasip1 and Cdh5, Rasip1 and Myl9a, Rasip1 and UCHD, as well as Cdh5 and Myl9a, were successfully replicated a minimum of five times in both wild-type and mutant embryos, where applicable, across independent experimental setups, yielding consistent results. Drug treatment, Opto-RhoA and expression of N-Rock1-Scarlet-Podxl1 were successfully replicated at least 3 times in independent experimental setups. Results were consistent across all replicates, confirming the robustness and reproducibility of the findings. |

| | |
|---|---|
| Randomization | Randomization was not applied in this study, as all experimental conditions (including sample preparation, imaging, and analysis) were conducted under identical settings for all mutant lines (rasip1, heg1, krit1, cdh5) and control samples. Given the nature of the experiments (e.g., immunostaining and live imaging), random assignment of samples was not deemed necessary. |
| Blinding | Blinding was not applied during this study because the nature of the experiments (e.g., immunostaining and live imaging with distinct markers) required knowledge of sample identity to correctly analyze specific structures such as apical compartments and cell-cell junctions. However, all experimental conditions were identical across groups to minimize bias. |

# Reporting for specific materials, systems and methods

We require information from authors about some types of materials, experimental systems and methods used in many studies. Here, indicate whether each material, system or method listed is relevant to your study. If you are not sure if a list item applies to your research, read the appropriate section before selecting a response.

## Materials & experimental systems

| n/a | Involved in the study |
|---|---|
| ☐ | ☒ Antibodies |
| ☒ | ☐ Eukaryotic cell lines |
| ☒ | ☐ Palaeontology and archaeology |
| ☐ | ☒ Animals and other organisms |
| ☒ | ☐ Clinical data |
| ☒ | ☐ Dual use research of concern |
| ☒ | ☐ Plants |

## Methods

| n/a | Involved in the study |
|---|---|
| ☒ | ☐ ChIP-seq |
| ☒ | ☐ Flow cytometry |
| ☒ | ☐ MRI-based neuroimaging |

## Antibodies

| | |
|---|---|
| Antibodies used | anti-zf-Podxl (1:200) (Herwig et al., 2011), rabbit anti-zf-Cdh5 (1:200) (Blum et al., 2008), guinea pigs anti-zf-Cdh5 (1:200) (Kotini et al., 2022), rabbit anti-Rasip1 (1:500) (Lee et al., 2021), mouse anti-human ZO-1 (Invitrogen, # 33-9100,1:500). Alexa 568 goat anti-rabbit immunoglobulin G (IgG) (1:1000; Thermo Fisher Scientific, A11011), Alexa 488 goat anti-mouse IgG (H+L) (1:1000; Thermo Fisher Scientific, A32723), Alexa 488 goat anti-guinea pig IgG (H+L) (1:1000; Thermo Fisher Scientific, A11073). |
| Validation | Antibody Validation Summary:<br>Anti-zf-Podxl (1:200): This antibody was previously validated and characterized in zebrafish by Herwig et al. (2011), where its specificity for zebrafish Podocalyxin (Podxl) was demonstrated using immunostaining techniques. The reference for its validation can be found in the original publication: Herwig et al., Current Biology (2011).<br>Rabbit anti-Rasip1 (1:500): The rabbit anti-Rasip1 antibody was validated by Lee et al. (2021), where it was confirmed to recognize Rasip1 in zebrafish using immunostaining. The full details can be referenced in: Lee et al., development (2021).<br>Rabbit anti-zf-Cdh5 (1:200): The antibody against zebrafish Cdh5 was validated by Blum et al. (2008), where it was shown to specifically recognize zebrafish VE-Cadherin (Cdh5) through immunofluorescence. Refer to: Blum et al., Developmental Biology (2008) for further details.<br>Guinea pig anti-zf-Cdh5 (1:200): It is validated through immunostaining in zebrafish endothelial cells and its specificity was confirmed in line with other reported Cdh5 antibodies. Refer to Kotini et al Cell Reports (2022).<br>Mouse anti-human ZO-1 (1:500, Invitrogen, #33-9100): The specificity of this antibody was verified by the manufacturer (Invitrogen) and has been widely used in both zebrafish and mammalian cells for recognizing zonula occludens 1 (ZO-1) protein in tight junctions. Validation information is available on the manufacturer's website and in product documentation.<br>For secondary antibodies:<br>Alexa 568 goat anti-rabbit IgG (1:1000, Thermo Fisher Scientific, A11011), Alexa 488 goat anti-mouse IgG (H+L) (1:1000, Thermo Fisher Scientific, A32723), and Alexa 488 goat anti-guinea pig IgG (H+L) (1:1000, Thermo Fisher Scientific, A11073) were validated by the manufacturer for use in fluorescence immunostaining. These are routinely used for detecting primary antibodies in a range of biological samples, and their validation is supported by extensive usage in literature. |

## Animals and other research organisms

Policy information about studies involving animals; ARRIVE guidelines recommended for reporting animal research, and Sex and Gender in Research

| | |
|---|---|
| Laboratory animals | Zebrafish embryos |
| Wild animals | This study did not involve the use of wild animals. |
| Reporting on sex | Sex was not considered in this study because zebrafish embryos used for the experiments were at developmental stages prior to sex differentiation. Therefore, the sex of the embryos was not determined or relevant to the outcomes. |
| Field-collected samples | This study did not involve the use of Field-collected samples |

| | |
|---|---|
| Ethics oversight | Zebrafish (Danio rerio) were maintained according to FELASA guidelines (Aleström et al., 2019). All experiments were performed in accordance with federal guidelines and were approved by the Kantonales Veterinäramt of Kanton Basel-Stadt (1027H, 1014HE2, 1014G). |

Note that full information on the approval of the study protocol must also be provided in the manuscript.

## Plants

| | |
|---|---|
| Seed stocks | *Report on the source of all seed stocks or other plant material used. If applicable, state the seed stock centre and catalogue number. If plant specimens were collected from the field, describe the collection location, date and sampling procedures.* |
| Novel plant genotypes | *Describe the methods by which all novel plant genotypes were produced. This includes those generated by transgenic approaches, gene editing, chemical/radiation-based mutagenesis and hybridization. For transgenic lines, describe the transformation method, the number of independent lines analyzed and the generation upon which experiments were performed. For gene-edited lines, describe the editor used, the endogenous sequence targeted for editing, the targeting guide RNA sequence (if applicable) and how the editor was applied.* |
| Authentication | *Describe any authentication procedures for each seed stock used or novel genotype generated. Describe any experiments used to assess the effect of a mutation and, where applicable, how potential secondary effects (e.g. second site T-DNA insertions, mosiacism, off-target gene editing) were examined.* |
